# Clustering by genetic ancestry using genome-wide SNP data

**DOI:** 10.1186/1471-2156-11-108

**Published:** 2010-12-09

**Authors:** Nadia Solovieff, Stephen W Hartley, Clinton T Baldwin, Thomas T Perls, Martin H Steinberg, Paola Sebastiani

**Affiliations:** 1 Department of Biostatistics, Boston University School of Public Health, Boston, MA, 02118, USA; 2 Center for Human Genetics, Boston University School of Medicine, Boston, MA, 02118, USA; 3 Geriatrics Division, Department of Medicine, Boston University School of Medicine, Boston, MA, 02118, USA; 4 Department of Medicine, Boston University School of Medicine, Boston, MA, 02118, USA

## Abstract

**Background:**

Population stratification can cause spurious associations in a genome-wide association study (GWAS), and occurs when differences in allele frequencies of single nucleotide polymorphisms (SNPs) are due to ancestral differences between cases and controls rather than the trait of interest. Principal components analysis (PCA) is the established approach to detect population substructure using genome-wide data and to adjust the genetic association for stratification by including the top principal components in the analysis. An alternative solution is genetic matching of cases and controls that requires, however, well defined population strata for appropriate selection of cases and controls.

**Results:**

We developed a novel algorithm to cluster individuals into groups with similar ancestral backgrounds based on the principal components computed by PCA. We demonstrate the effectiveness of our algorithm in real and simulated data, and show that matching cases and controls using the clusters assigned by the algorithm substantially reduces population stratification bias. Through simulation we show that the power of our method is higher than adjustment for PCs in certain situations.

**Conclusions:**

In addition to reducing population stratification bias and improving power, matching creates a clean dataset free of population stratification which can then be used to build prediction models without including variables to adjust for ancestry. The cluster assignments also allow for the estimation of genetic heterogeneity by examining cluster specific effects.

## Background

In GWAS, varying ancestral backgrounds lead to population stratification inflating the type I error rate [1,2]. Even European Americans are affected by population stratification bias [3].

PCA [4], spectral graph theory [5], structured association analysis [6,7] and genomic control [8] can detect underlying population substructure with SNP data. Investigators typically use PCA to detect population structure and then adjust the estimates of genetic effects for the top number of principal components (PCs) to control for population stratification bias [4]. Genomic control divides the test statistic for each SNP by the genomic control inflation factor (λ), defined as the observed median test statistic across all tests genome-wide divided by the expected median test statistic. The test statistic for each SNP is divided by the same value, irrespective of whether the particular SNP is structured, and thus results in a loss of power [4]. A wide range of alternative methods have been proposed to adjust for population stratification by applying an adjustment to the test statistic, using permutation tests or by performing stratified analyses [9-11].

Alternatively, population stratification can be obviated by matching cases and control with respect to genetic ancestry [12]. Matching does not require the investigator to account for population stratification bias by adjusting for PCs or dividing test statistics by the genomic control inflation factor. Guan et al [13] match cases and controls using a genetic similarity score computed directly from genotype data as a weighted identity by state estimate.

We propose a different algorithm in which we identify clusters of varying genetic ancestry using the results of PCA from genome-wide data. The algorithm includes a novel approach to choosing the appropriate number of informative PCs, a clustering step to group subjects into clusters of genetic diversity and a novel "scoring index" (SI) to choose the appropriate number of clusters. Luca et al [14] implemented a similar algorithm for matching cases and controls with the main difference being that parametric tests that may not be robust to departures from model assumptions are used while our algorithm is non-parametric and does not require strict assumptions.

We test the sensitivity and specificity of our algorithm on simulated data and provide applications on African populations from the Human Genome Diversity Project [15] and a cohort of centenarians with European ancestry [16]. We demonstrate that by matching cases and controls within each cluster the inflation in test statistics caused by population stratification bias substantially decreases as compared to unmatched cases and controls and in certain situations matching is more powerful than adjusting for the top number of PCs. More importantly, our method allows for the creation of a dataset free of population stratification which can then be used for prediction modeling [17]. Prediction models containing principal components as covariates are study specific and cannot easily be generalized to a different study in which the values of the PCs are unknown. The clusters produced by the algorithm also allow for the exploration of locus heterogeneity and we provide an example of a SNP in *APOE *in which the odds ratio varies widely across ethnic groups in Europe.

## Results

### Algorithm

**Figure 1:**
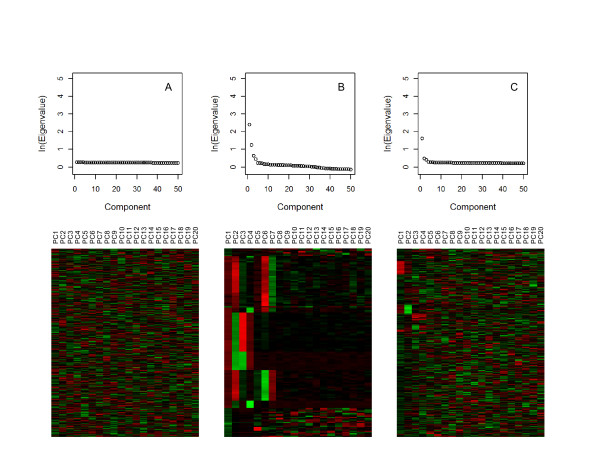
**Scree Plot and Heatmap of Top 20 PCs**: The scree plots and heatmaps are presented for the simulated data with no structure (left), HGDP Africans (middle), NECS (right). The scree plot graphs the natural logarithm of the eigenvalue (y-axis) versus the component number (x-axis). A kink is not observed in the scree plot for the simulated data with no population substructure (a). For the HGDP Africans (b) and the NECS (c) the scree plot identifies a kink at the 5^th ^and 4^th ^PCs, respectively. In the heatmaps, each column is a PC and each row is an individual. Original PCs are standardized by row and the color intensities correspond to the standardized value of the PC for each individual (green: higher than average, red: lower average) and are sorted by the corresponding eigenvalues, and rows are sorted by hierarchical clustering. While no pattern is found in the heatmap of the simulated example without population structure, a distinct pattern is observed for the HGDP Africans and the NECS. The pattern in the HGDP Africans is the most distinct since these populations are well defined and are very different from each other. The pattern for the NECS is more subtle because the variability in subjects of European ancestry is much lower than in Africans. However, one can still observe variability beyond the first 2 principal components.

The algorithm that we developed consists of the following three components: 1) selection of informative PCs for the cluster analysis; 2) clustering to discover population substructure; 3) a novel score to automatically select the best number of clusters that satisfy a variety of criteria. We select the informative PCs by visually inspecting a heatmap and scree plot of the top PCs (Figure 1). A heatmap displays the top PCs in the columns and the subjects in the rows. The intensity of the color corresponds to the value of the PC and the subjects are reordered so that individuals with similar PC values are displayed next to each other in the heatmap. The heatmap allows one to visually inspect the joint pattern produced by a set of PCs and to choose the informative PCs. The scree plot graphs the natural logarithm of the eigenvalue versus the component number. The informative number of PC is identified by a "kink" in the plot after which a straight line is observed. Based on the top PCs, we cluster individuals using k-means clustering. K-means clustering assigns subjects to a pre-specified number of clusters by maximizing the distance between subjects in different clusters. Distance is defined as the Euclidean distance between subjects with respect to their top PCs. Since k-means does not provide a metric for choosing the appropriate number of clusters, we developed an algorithm and scoring index to identify the optimal number of clusters. The algorithm performs M executions of k-means clustering for each cluster size, k = 2,3,...K, and computes a measure of accuracy, stability and between cluster distance for each execution and cluster size. We use these 3 measures because an optimal cluster assignment should accurately assign individuals based on the PCs, should be consistent from execution to execution for a particular number of clusters and should also maximize the distance between individuals in different clusters. We compare these three measures computed in the data to those expected under random cluster allocation using permutation analysis, and summarize the gain of accuracy, stability and distance into a score. The derivation of the components is described in detail in the methods. In the following sections we describe the results of the experiments we conducted to evaluate the properties of the algorithm.

### Simulations

We first simulated genotype data containing no population substructure (see Methods). The heatmap of the top PCs shows no distinct pattern and the scree plot is flat (Figure 1A) suggesting that the data do not contain substructure and clustering is unnecessary. To assess the sensitivity of the method, we simulated genotype data with the number of clusters ranging from 2 to 10 clusters under two scenarios, equal and unequal sample sizes across clusters (see Methods). The power of the scoring index to identify the correct cluster size and allocate subjects to the correct cluster was greater than 95% in most cases and in some cases 100% (Table 1). When the maximum scoring index was used to identify the cluster size, the power was low for a cluster size of 2 with equal sample sizes (78%) and a cluster size of 2 and 3 with unequal sample sizes (56%, 64%). In these situations the scoring index remained relatively constant across several cluster sizes and the maximum SI tended to overestimate the true number of clusters. However, using the optimal SI increased the power to 100% for a cluster size of 2 with equal sample sizes and 99% and 97% for a cluster size of 2 and 3 with unequal sample sizes. The optimal SI is defined as the smallest cluster size for which the estimated SI falls within the 95% confidence interval of the maximum SI.

**Table 1:** Power of Scoring Index

	Scenario 1: Equal N		Scenario 2: Unequal N	
K	Max SI	Optimal SI	Max SI	Optimal SI
2	0.78	1	0.56	0.99
3	0.97	1	0.64	0.97
4	0.99	1	0.86	0.99
5	1	1	0.99	1
6	0.99	1	0.99	0.99
7	1	1	0.99	0.99
8	1	1	0.99	0.99
9	1	1	0.99	0.99
10	1	1	1	1

We report the proportion of datasets for which the correct number of clusters is identified and the subjects are correctly allocated their respective cluster. The maximum SI corresponds to the cluster size at which the SI is maximized and the optimal SI corresponds to the smallest cluster size for which the SI falls within the 95% CI of the maximum SI.

### Real Data with African Ancestry

**Figure 2:**
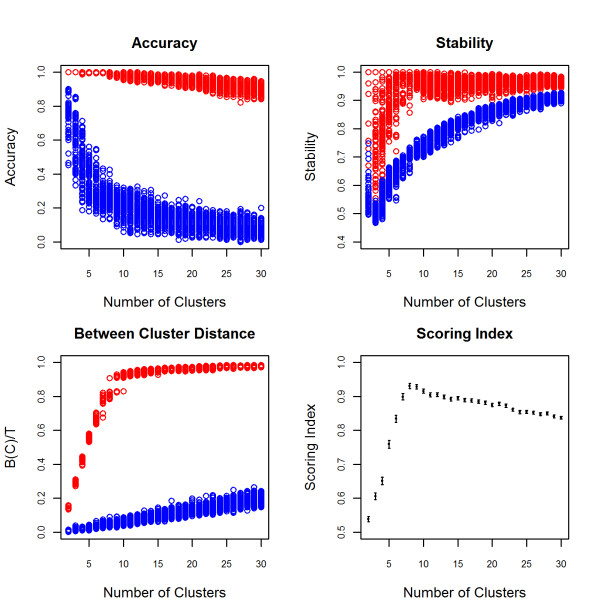
**Accuracy, Stability, Between Cluster Distance and Scoring Index (SI) for HGDP Africans**. Accuracy, stability and between cluster distance on the k-means cluster assignments are displayed in red and the measures on the permuted cluster assignments are displayed in blue. The SI averages the relative gain in the accuracy, stability and between cluster distances and maximizes at 8 clusters (0.931, CI: 0.923, 0.938)). Note that the graphical display of accuracy, stability and distance shows that none of the score components would be sufficient to identify the correct number of clusters. For example, high accuracy is not sufficient to conclude that the clustering is optimal. In this example, the accuracy is nearly perfect when the number of clusters is less than 7 suggesting that any number of clusters less than 7 is equally optimal, however these numbers are not all equally optimal as demonstrated by the measures of stability and between cluster distances that continually increase.

**Figure 3:**
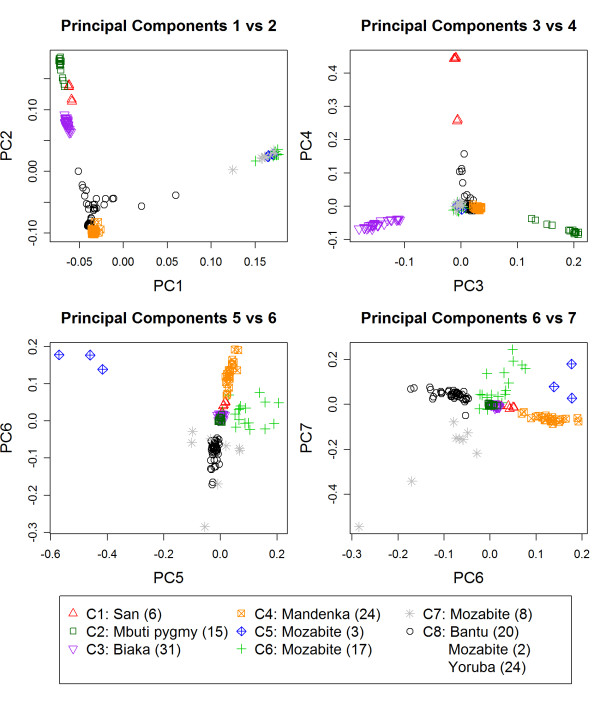
**Pairwise PC Plots of HGDP Africans Colored by Cluster Assignment**. The legend reports the cluster assignment, population and sample size for each cluster. The Biaka, Mandenka, Mbuti pygmy and San are separated into the first 4 clusters. The eighth cluster (black) contains the Yorubans, Bantu and 2 subjects from the Mozabite population and these subjects cluster together in each of the above plots indicating that these cannot be differentiated based on the first 7 PCs. The remaining Mozabite are split between clusters 5-7 and show variability in PC5, PC6 and PC7 suggesting that these individuals are more heterogeneous than the other African populations.

We then tested the sensitivity of our algorithm to group real data from individuals with known ancestry from 7 African populations in the Human Genome Diversity Panel. The heatmap identified the top 7 PCs as most informative while the scree plot identified the top 5 PCs (Figure 1). Since the pattern for the top 7 PCs is quite strong in the heatmap, we used the top 7 PCs instead of only the top 5 for clustering. The SI identified 8 clusters as the optimal cluster size (Figure 2). The Biaka, Mandenka, Mbuti Pygmy and San populations are assigned to their own cluster. The Yorubans are clustered with the Bantu and 2 of the Mozabite subjects in the 8^th ^cluster and show a stronger similarity with each other than with the other Africans in this analysis. The plots of the PCs support the clustering of these subjects (Figure 3) and are consistent with the origin of the Bantu near the southern boundary of modern Nigeria and Cameroon and the known genetic similarity between Bantu and Yorubans [18,19]. In the plot of PC6 and PC7 (Figure 3) we see a distinct separation of the Mandenka from the Yorubans and Bantu which indicates that these PCs are important to increase the sensitivity of clustering. The remaining Mozabite subjects were split among 3 clusters. This genetic diversity is consistent with the history of the Mozabites and recent studies that reveal genetic ancestry from sub-Saharan Africa, the Middle East and Europe [18]. Previously reported analyses of these data that were based on inspection of the first two PCs failed to detect this level of diversity based on purely genetic data. Patterson et al [19] could only detect differences between San and Bantu/Yorubans combined. Our analysis shows that a larger number of PCs is necessary to detect finer population structure that could introduce confounding in GWAS and confirms the utility of the heatmap plot of PCs.

### Real Data with European Ancestry

**Figure 4:**
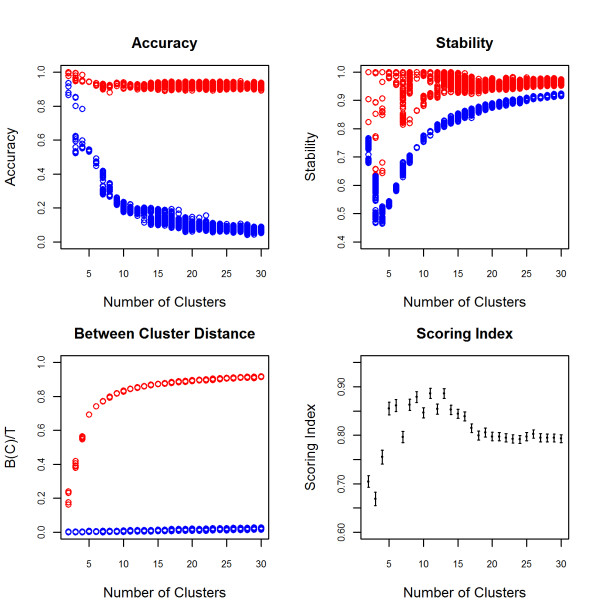
**Accuracy, Stability, Between Cluster Distance and Scoring Index for NECS**. Accuracy, stability and between cluster distance on the k-means cluster assignments are displayed in red and the measures on the permuted cluster assignments are displayed in blue. The scoring index maximizes at 11 clusters (SI = 0.886, 95% CI: (0.876, 0.896)). The mean score for 9 clusters (0.879) falls within the 95% confidence interval of 11 clusters and thus is as good as 11 clusters but is more parsimonious. and is also the optimal solution. In general, we expect the permuted stability to increase with an increasing number of clusters because a larger number of pairs will be consistently assigned to different clusters since there are more available clusters. However, note the peculiar trend of cluster stability in this example: the permuted stability decreases when moving from k = 2 to 4 clusters and then gradually increases. This occurs because one cluster contains the majority of the subjects for these clusters sizes and thus the number of pairs of subjects assigned to the same cluster, by chance, is much higher than in the case where there are equal sample sizes in each cluster. Thus it is important to account for the permuted measurements when selecting the optimal number of clusters.

**Figure 5:**
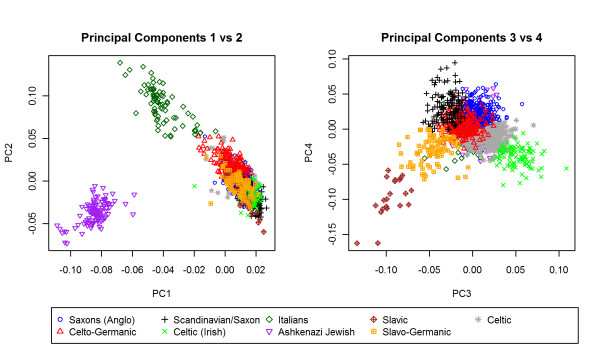
**Plot of First 4 PCs of NECS Colored by Cluster Allocation**. PC1 and PC2 clearly separate the subjects of Jewish descent, shown in purple, from the rest of the subjects and create a northwestern to southeastern European cline. PC3 and PC4 capture further variability within European ethnic groups. The cluster assignments found by the algorithm closely correspond to established ethnic groups across Europe. Based on partial information (birth places and native language of grandparents) of the subjects' ancestry, the clusters were labeled according to the most common ethnic group in the region [37].

In a cohort of 1051 centenarians and 290 controls from the New England Centenarian Study, the algorithm detected many specific European ethnic groups. The heatmap and the scree plot identified the top 4 PCs as informative (Figure 1) and the algorithm identified 9 clusters (Figure 4). Based on partial information of ancestry (birth places and native language of grandparents) for the subjects in the NECS, we found that the 9 clusters correlated with distinct ethnic groups in Europe (Figure 5) demonstrating that the clustering algorithm can detect fine distinctions among subjects of European descent. The population substructure in subjects of European descent is typically identified as the pattern formed by PC1 and PC2 in Figure 5 that identifies Ashkenazi Jews and a Northwest to Southeast cline across Europe [3,20]. The pattern formed by PC3 and PC4, generally not reported, also correlates clearly with specific ethnic groups in Europe such as the Anglo-Saxons, Celto-Germanic, Scandinavians, Saxons, Irish-Celtic, Italians, Ashkenazi Jewish, Slavic, Slavo-Germanic and Celtic.

### Effect of Matching on the Power of a GWAS

**Figure 6:**
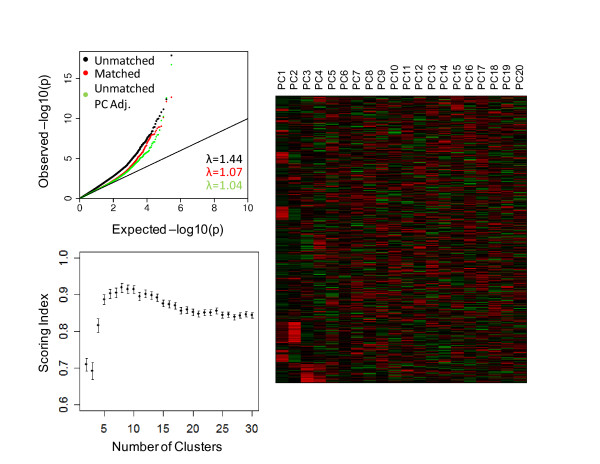
**Q-Q Plot of GWA results, Scoring Index and Heatmap for NECS with Matched and Unmatched Controls**. The QQ plot (top left) shows the substantial decrease of the inflation in the GWA results when controls are matched to cases within each cluster with the genomic control factor, λ, decreasing from 1.44 to 1.07. When adjusting for the top 4 PCs in the unmatched analysis, λ decreases to 1.04, however, many of the top associations are missed (Figure 7). The scoring index (bottom left) for the combined analysis of the NECS and controls from the Illumina database maximized and optimized at 8 clusters (SI = 0.919, 95% CI: (0.907, 0.931)). The heatmap displays a pattern for the top 4 PCs.

**Figure 7:**
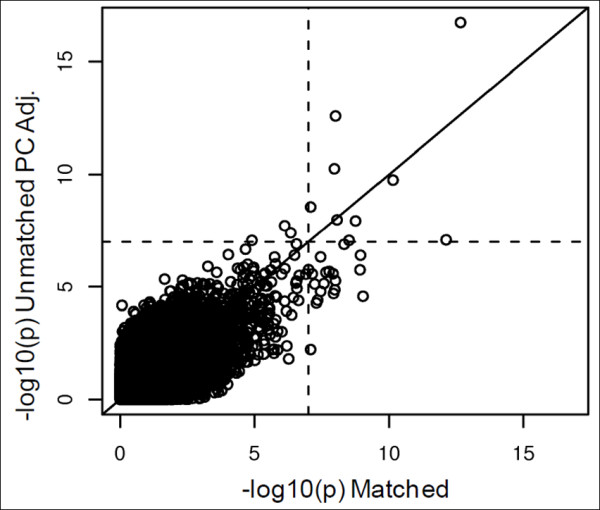
**GWAS Results of EL for Matched and Unmatched Subjects**. The x-axis reports the -log10 p-values for the GWAS in which subjects were matched based on 8 clusters. The y-axis reports the -log10 p-values for the GWAS controlling for the top 4 PCs in which an equal number of subjects were randomly chosen from the Illumina controls. The dotted line is drawn at a value of 7. The SNPs in the bottom right hand rectangle are SNPs which would be selected as the top SNPs in the matched and would be missed in the unmatched PC adjusted analysis. There are 19 SNPs with a p-value less than 10E-07 for the matched analysis for which the p-value is greater than 10E-07 for the unmatched PC adjusted analysis.

Understanding the genetic diversity of the NECS allows one to select genetically matched controls and reduce population stratification bias. For example, in our GWAS of exceptional longevity (EL) [17] we set out to expand the NECS control sample by drawing cluster-matched subjects from a sample of approximately 3,600 subjects from the Illumina database. We conducted a combined PCA of the NECS centenarians and controls, and ~3,600 Illumina controls and the heatmap of the top 20 PCs suggested that the top 4 PCs are the most informative (Figure 6). The clustering algorithm identified 8 clusters as the optimal solution (SI = 0.919, 95% CI: (0.907, 0.931)). We matched cases to controls within each of the 8 clusters to balance the overall proportion of cases and controls across the clusters, resulting in the addition of 745 Illumina controls to the existing NECS controls set. We then performed two sets of GWAS for EL, one with the matched data and a second analysis with an equal number of randomly selected Illumina controls, using additive models. The QQ plot in Figure 6 displays the results for SNPs genome-wide and demonstrates the striking reduction in the inflation of test statistics for the matched controls as compared with the unmatched controls. The genomic control factor λ decreases from 1.44 for the unmatched set to 1.07 for the matched set. Matching does not require the investigator to account for population stratification bias by adjusting for PCs or by dividing test statistics with the genomic control inflation factor which can decrease the power of detecting true associations [4]. For example, an unmatched PC-adjusted analysis with the same number of controls randomly chosen from the Illumina database would lose almost all of the top associations suggesting a loss in power. (Figure 7) Furthermore, in the QQ plot (Figure 6) the tail of the distribution of p-values for the adjusted unmatched GWAS is lower than the tail for the matched GWAS which again suggests a loss in power. The genomic control factor for the unmatched PC adjusted analysis (λ = 1.04) is slightly lower than for the matched analysis, however the difference is not substantial.

### Effect of Matching on Power

**Figure 8:**
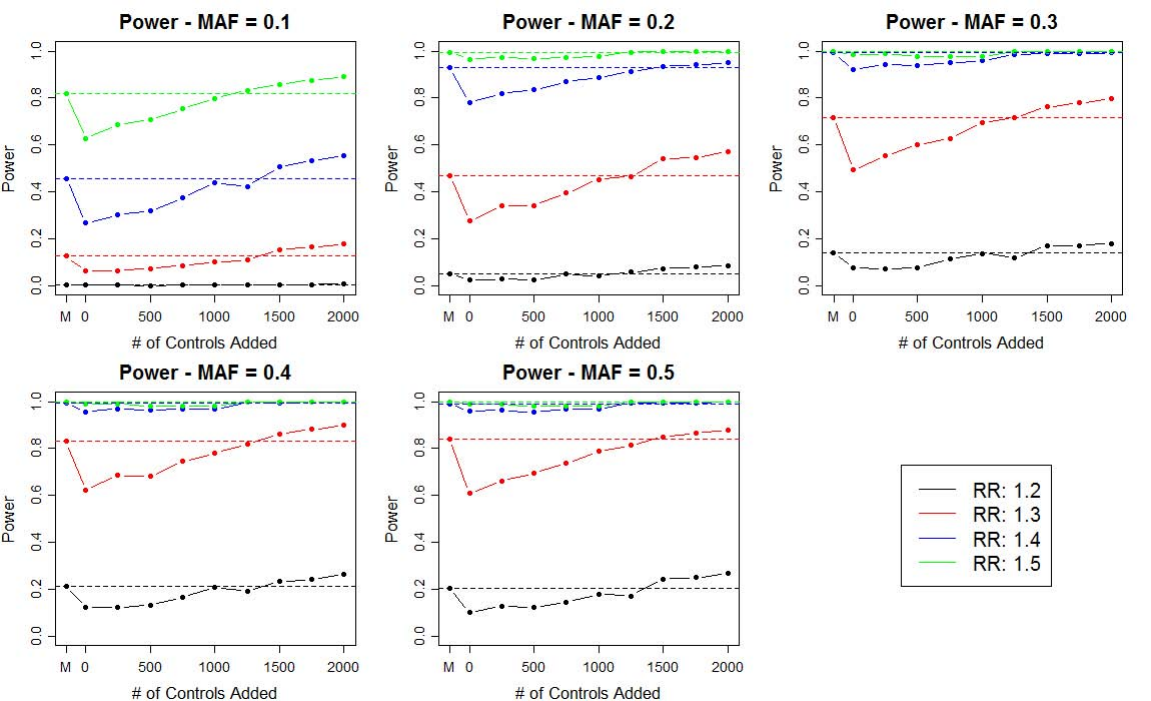
**Power of Matched versus Unmatched PC Adjusted GWAS**. We compared the power of a matched GWAS (2000 cases/2000 controls) to an unmatched PC adjusted GWAS with a varying number of controls (2000 to 4000). The x-axis reports the number of controls added to the unmatched control group (n = 2000) and the letter M corresponds to the matched GWAS. Power was assessed for a MAF ranging between 0.1 to 0.5 and a relative risk (RR) ranging from 1.2 to 1.5. In all scenarios the matched analysis is more powerful than the unmatched analysis when the sample is the same for both analyses. As we add more controls to the unmatched analysis, the power increases and surpasses the power of the matched analysis when 1000 to 1500 additional controls are added. Note that the power is adjusted for the varying false positive rate across scenarios.

To investigate the effect of matching on the power of a GWAS, we simulated genotype data to compare the power and false positive rate of a GWAS in which we match cases and controls and a GWAS in which the subjects are unmatched but the analysis is adjusted for the top 10 PCs (see Methods). The false positive rate across all scenarios was close to the nominal level (Table 2). The GWAS using the matched subjects was more powerful than the PC adjusted unmatched analysis with the same sample size (Figure 8). The gain in power for matched analysis ranged from 1.0% to 23.4%. When matching is performed, a number of controls are excluded due to the inability to match them. To examine the effect of adding in all controls versus using a smaller pool of matched controls, we added a varying number of controls to the unmatched analysis (see Methods). As expected the power of the PC adjusted unmatched analysis increases as more controls are added and surpasses the power of the matched analysis when 1000 to 1500 subjects are added (Figure 8). This result suggests that using all controls and adjusting for PCs as opposed to matching will become more powerful if the number of unmatched controls is substantially larger than the number of matched controls.

**Table 2:** False Positive Rate of Matched and PC adjusted Unmatched GWAS

Scenario	Cases/Controls	False Positive Rate*
Matched	2000/2000	5.00E-07
Unmatched + PCs	2000/2000	8.00E-07
Unmatched + PCs	2000/2250	9.00E-07
Unmatched + PCs	2000/2500	1.30E-06
Unmatched + PCs	2000/2750	1.20E-06
Unmatched + PCs	2000/3000	9.00E-07
Unmatched + PCs	2000/3250	1.00E-06
Unmatched + PCs	2000/3500	8.00E-07
Unmatched + PCs	2000/3750	9.00E-07
Unmatched + PCs	2000/4000	8.00E-07

*False positive rate with a p-value cut-off of 1E-06

We report the false positive rate of a matched GWAS and compare it to PC-adjusted unmatched GWASs with varying number of controls. The false positive rate is computed using a p-value cut-off of 1E-06.

### Locus Heterogeneity

**Figure 9:**
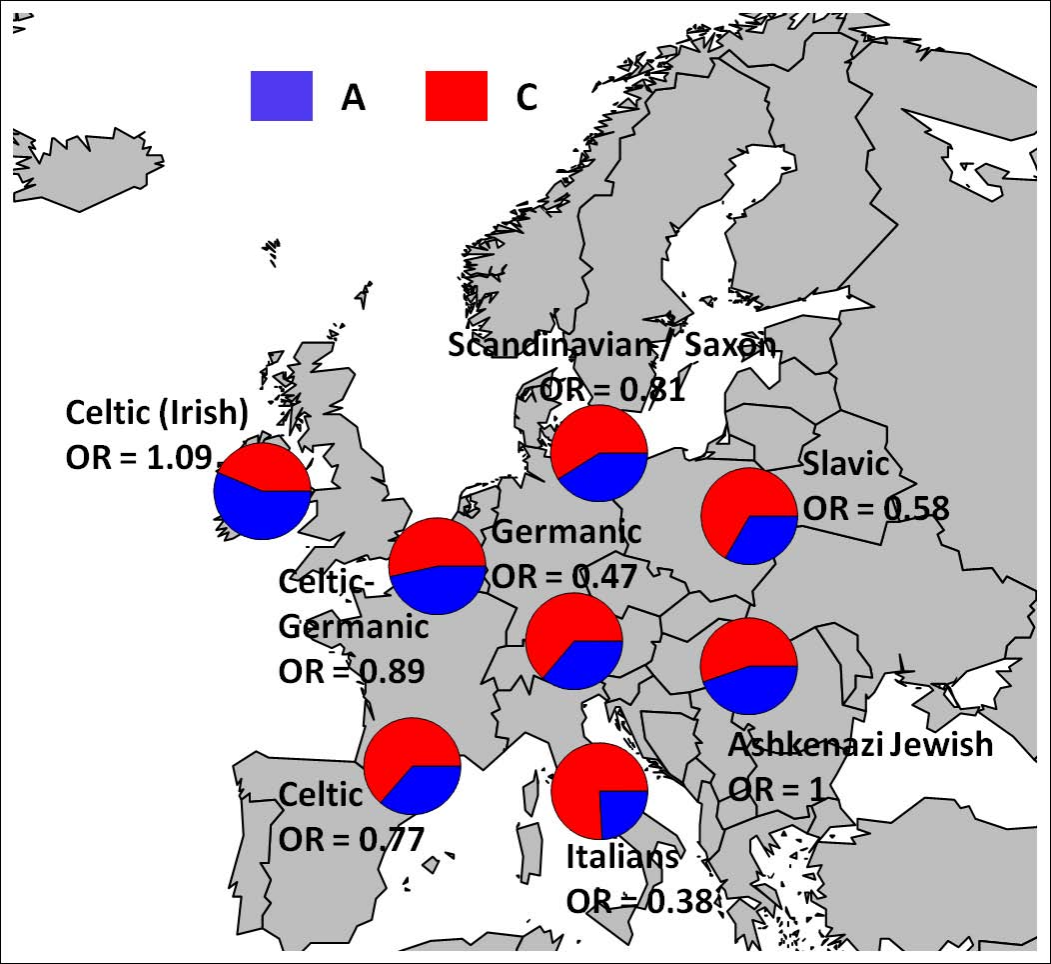
**Frequency of rs405509 in Centenarians in 8 Clusters**. The allele frequencies of rs4005509, a SNP near *APOE*, vary greatly between the 8 clusters of centenarians identified in the analysis of the NECS and Illumina subjects. The odds ratio for each cluster is calculated using a separate logistic regression model of exceptional longevity and the SNP genotype coded as the number of A alleles. The odds ratios are quite different among various ethnic groups indicating that *APOE *may have a different impact in different ethnic groups.

Breaking the subjects into clusters also has the advantage of examining cluster specific allele frequencies and genetic effects. As an example, the allele frequencies for rs405509, a SNP near *APOE *known to be associated with exceptional longevity and other age-related diseases [21,23], varies greatly among European ethnic groups (Figure 9). Interestingly the association between EL and this SNP also varies between clusters with odds ratios ranging from 0.38 in the Italians to 1.09 in the Irish Celtics. (Figure 9) Small differences in allele frequencies can affect the power to detect a true main effect when the SNP is part of a SNP- SNP interaction [24] and thus close examination of cluster specific allele frequencies between discovery and replication sets can be useful for replication.

## Discussion

Our analyses suggest that the algorithm works well in simulated datasets and in real data of Africans and Caucasians of European ancestry and can reduce or eliminate population stratification. Choosing the appropriate number of PCs for clustering is a critical choice. We chose the number of PCs by evaluating patterns observed in a heatmap and in a scree plot; although subjective, in many situations a clear choice is evident. We emphasize that investigators should carefully examine the results of the PCA prior to clustering to ensure that population substructure in fact exists in the data. If population substructure is absent, clustering is unnecessary and will lead to overfitting. Although we use k-means clustering in our algorithm [25], alternative techniques including k-medoids, hierarchical clustering, and model based clustering could be used. Model based clustering requires parametric assumptions about the distribution of the data and it can outperform k-means clustering when the assumptions are correct but perform poorly when they are not.

**Figure 10:**
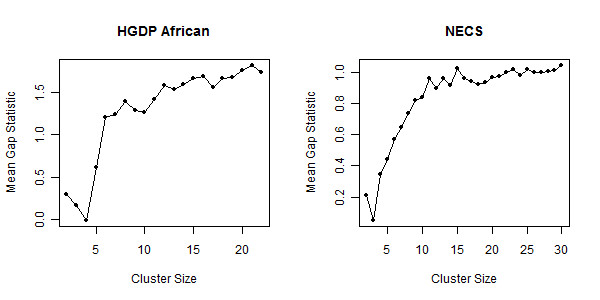
**Gap Statistic Across Varying Cluster Sizes for HGDP Africans and NECS**. The graph plots the mean gap statistic (see Methods) at each cluster size. The gap statistic for the HGDP Africans steadily increases to 21 clusters at which point the statistic cannot be computed due to singleton clusters. For the NECS, the statistic again increases with an increasing cluster size and does not reach a point after which the statistic continuously decreases. In both situations choosing an optimal cluster size is not obvious.

The SI provides a novel statistic for choosing the best number of clusters by combining measures of accuracy, stability and between cluster distances. Maximizing only one or two of these measures is insufficient for choosing an optimal number of clusters. For example, the clustering may be accurate and stable for a given number of clusters, however there may be a different number of clusters for which the accuracy and stability are comparable but the between cluster distance is better. Some advocate choosing the number of clusters at which a "kink" occurs in the between cluster distance since the measure will increase by a larger amount when informative clusters are created and will increase by a smaller amount when the clustering method begins to create uninformative clusters [25]. However, one does not always observe a distinct "kink" (see Figure 4). An alternative statistic called the gap statistic [26] monitors the within cluster distance, compares it to a null distribution and chooses the number of clusters for which the deviation of the within cluster distance from the null distribution is maximized. In practice, a maximum may never be attained for a reasonable range of cluster sizes as demonstrated in the examples of the HGDP Africans and NECS (Figure 10).

We have shown that matching is an effective method to reduce the inflation in test statistics due to population stratification both in simulated cases and real data. Matching in some situations is more powerful than using an unmatched dataset and adjusting for PCs. Our method also allows one to create a dataset free of population stratification which can be used to build prediction models. We created a clean dataset for the NECS which was used to build a model of exceptional longevity and we replicated the model in an independent study with high accuracy [17]. The advantage of this approach is that we did not need to account for population substructure in the model and were able to generalize it to an independent study. Including PCs in a prediction model causes the model to be study specific and may be difficult to reproduce.

Furthermore as the availability of external controls increases through organizations like dbSNP, investigators can use this method to match the controls to cases with respect to ancestry and reduce the issue of population stratification. Zhuang et al [27] show that expanding the control group can improve power. Of course, investigators must take precautions to avoid introducing other forms of bias due to the use of external controls.

As next generation sequencing and the discovery and analysis of rare variants continue to emerge, population stratification will remain an obstacle. Our algorithm can easily be implemented to match cases and controls when selecting subjects for sequencing and insure that the selection adequately represents all ethnicities in the sample thus increasing the chance of finding true novel polymorphisms.

## Conclusions

We developed an algorithm to cluster individuals into ethnic groups based on PCs computed from SNP data. We showed that the algorithm works well in real data of Africans and Caucasians with European ancestry and also in simulated data. The cluster assignments can be used to effectively match cases and controls in GWAS to reduce and even eliminate population stratification bias. Matching can also aid in genetic risk prediction models by creating a dataset free of population stratification. Furthermore, the cluster assignments can be used to study locus heterogeneity and identify SNPs and genes that have a different effect on the phenotype in different ethnic groups.

## Methods

### Simulated Datasets

#### Dataset with No Population Substructure

To test the algorithm on a dataset containing no population substructure, we simulated 100,000 SNPs in linkage equilibrium for 2000 individuals with no underlying pattern. The allele frequencies for each SNP were randomly simulated with values ranging between 0.05 and 0.95. Genotype frequencies were computed from the allele frequencies assuming Hardy Weinberg equilibrium and individuals were randomly assigned genotypes according to the genotype frequencies.

#### Dataset with Known Structure

We simulated genotype data in which the number of clusters ranged from k = 2 to 10. Each dataset contained 1000 individuals and 10,000 SNP in linkage equilibrium. Each cluster was simulated with Fst = 0.01 corresponding to differences observed among divergent European populations [28]. For each SNP the founder allele frequency, p, was selected from a uniform(0.05,0.50) distribution, the allele frequency for each cluster, p_k_, was selected from a beta distribution with shape parameter α = p(1-Fst)/Fst and β = (1-p)*(1-Fst)/Fst and the genotype frequencies were computed as (Fst(1-p_k_) + (1-Fst)(1- p_k_)^2^, 2(1-Fst) p_k_(1- p_k_), Fst p_k _+ (1-Fst) p_k_^2^) [29,30]. In scenario 1 we split the subjects equally among the clusters, rounding up to the nearest integer. In scenario 2, the sample size within each cluster was randomly chosen with the requirement that each cluster must contain at least 5% of the total sample size. In each scenario, 900 dataset were simulated (100 for each cluster size k). For each dataset, a PCA was performed and the clustering algorithm was used to cluster individuals into groups. The sensitivity of the algorithm is measured by the proportion of datasets in which the cluster size is correctly identified by the scoring index and the algorithm correctly allocates all the subjects to their respective cluster.

#### Power Analysis

To simulate a scenario in which subjects are matched with respect to their ancestry, we simulated 2000 cases and 2000 controls from 4 underlying clusters with the probability (0.4, 0.3, 0.2, 0.1) of being in clusters 1, 2, 3 or 4, respectively. In the unmatched scenario with the same sample size, we generated 2000 cases from 4 clusters with probability (0.4, 0.3, 0.2, 0.1) and 2000 controls with probability (0.1, 0.2, 0.3, 0.4). To examine the effect of adding controls to the unmatched scenario, we simulated a pool of 2000 extra controls from 4 clusters with probability (0.1, 0.2, 0.3, 0.4). We then randomly added a varying number of controls (250, 500, 750, 1000, 1250, 1500, 1750, 2000) to the unmatched scenario. 10,000 SNPs in linkage equilibrium unrelated to disease status were simulated according to the model described in the previous section and were used to estimate the false positive rate. Twenty-four causal SNPs were generated with relative risks (R) of 1.2, 1.3, 1.4, 1.5 and minor allele frequencies (p) of 0.1, 0.2, 0.3, 0.4 and 0.5. The genotype frequencies for the controls were simulated as previously described. For the cases the genotype frequencies were computed as (Fst(1-p_k_) + (1-Fst)(1- p_k_)^2^, 2R(1-Fst) p_k _(1- p_k_), R^2^(Fst p_k _+ (1-Fst) p_k_)) where R corresponds to the relative risk. These frequencies were scaled by the sum of the 3 genotype frequencies so that the probabilities added up to 1.

### Real Datasets

#### Human Genome Diversity Project (HGDP) Africans

We tested our algorithm on 151 individuals from the 7 distinct African populations in the Human Genome Diversity Project (Bantu, Biaka, Mandenka, Mbuti pygmy, Mozabite, San, Yoruba) whose ancestry we knew a priori [15] (Table 3). Data were downloaded from the Illumina iControlDB database. We performed PCA on 263,722 autosomal SNPs with a call rate greater than 95% and minor allele frequency greater than 5% and applied our algorithm to the PCs. All subjects had a call rate greater than 93%.

**Table 3:** Populations and Studies

Population/Dataset	N
Human Genome Diversity Project: African	
Bantu	20
Biaka	31
Mandenka	24
Mbuti pygmy	15
Mozabite	30
San	6
Yoruba	24
New England Centenarian Study (NECS)	1341
Illumina Database	3613

#### New England Centenarian Study (NECS) set

A subset of subjects from the New England Centenarian Study containing 1051 cases and 290 controls was used to test the algorithm. This study was approved by the Boston University Institutional Review Board. The initial PCA, containing 298,734 SNPs, identified many chromosomal regions with elevated SNP weights due to strong LD in those regions. We therefore removed SNPs in strong LD using the program PLINK [31] with a SNP window of 50, sliding window of 5 SNPs and removed 1 SNP from each pair of SNPs with r^2 ^> 0.30. The final dataset contained 96,457 SNPs with a call rate greater than 95% and minor allele frequency greater than 5%. PCs from final dataset did not have elevated SNP weights for any chromosomal regions for the top 20 PCs. We found that setting the r^2 ^threshold higher than 0.30 resulted in elevated SNP weights in many chromosomal regions. All subjects had a call rate greater than 93%.

#### NECS and Illumina Control set

3,613 controls labeled as Caucasian were selected from the Illumina control database (iControlDB) and combined with the NECS controls. There were 298,734 SNPs common to the NECS and Illumina datasets that had a SNP call rate > 0.95 and MAF > 0.05. SNPs in strong LD were removed using the program PLINK with a SNP window of 50 and sliding window of 5 SNPs and we removed 1 SNP from each pair of SNP with r^2 ^> 0.30 leaving 97,508 SNPs for the analysis. A PCA was performed on the combined data. All subjects had a call rate greater than 93%. To check for differences between the two datasets, we compared the MAFs between the NECS dataset and Illumina controls. All SNPs had less than a 10% difference in MAFs between the two datasets and 99.9% of the SNPs had less than a 5% difference in MAF. We also compared the PCs using only the NECS dataset with the PCs using the NECS and Illumina datasets. We found that the top 4 PCs which were used for clustering were strongly correlated among the NECS between the two PCAs showing that the addition of the Illumina controls did not bias the results of the PCA. The correlation coefficients (Spearman) were 0.98, -0.89, 0.87 and 0.76 for the 1st-4th PCs, respectively.

#### Principal component analysis

In all applications we used the principal components analysis implemented in the software smartpca [4] to detect population substructure among individuals with genome-wide data.

### Algorithm

The algorithm consists of the following components: 1) selection of informative PCs for the cluster analysis; 2) clustering to discover population substructure; 3) a novel score to select the best number of clusters that satisfy a variety of criteria.

### How Many Informative PCs?

The Tracy-Widom statistic can be used to identify the ancestrally informative principal components. However this statistic is very sensitive to the inclusion of SNPs in linkage disequilibrium (LD) and tends to identify a larger number of PCs [19,32]. Typically, investigators use the first 10 PCs although this choice is somewhat arbitrary. We identify informative PCs for the algorithm by displaying the results of the PCA in a heatmap and by the use of a scree plot [33] which plots the natural logarithm of the eigenvalues.

We display the top 20 principal components in a heatmap in which the color of each cell (i, j) represents the value of the principal component in column j for the subject in row i, standardized by row. We order the rows using hierarchical clustering so that individuals with similar values for PCs are arranged next to each other. The heatmap highlights the most evident groups and the number of PCs that determine these groups. Because the interpretation of the visual display is subjective, we also use a scree plot of the natural logarithm of the eigenvalues to identify the important PCs. A scree plot graphs the log of the eigenvalue for each PC versus the PC numbers, and the appropriate number of PCs is identified by a "kink" in the plot after which we observe a relatively straight line (Figure 1).

### K-Means Clustering of the Most Informative PCs

We use k-means clustering to group subjects into K groups, for a fixed K. K-means assigns subjects to groups by minimizing the distance between subjects within each cluster (within cluster distance W(C)) or equivalently maximizing the distance between subjects in different clusters (between cluster distance B(C)). The overall "within cluster distance" W(C) is the sum of the distances between subjects allocated to the same clusters:

$$W(C)=\frac{1}{2}\sum_{k=1}^{K}\sum_{C(i)=k}\sum_{C(i')=k}d(x_{i},x_{i'})$$

and the "between clusters distance" B(C) is the distance between subjects allocated to different clusters, which is given by the difference between the total distance between subjects and the W(C):

$$\text{Total}=\frac{1}{2}\sum_{i=1}^{N}\sum_{i'=1}^{N}d(x_{i},x_{i'})\text{ and B}\left(\text{C}\right)=\text{Total}-\text{W}\left(\text{C}\right).$$

The distance between subjects within the same cluster k is defined as:

$$D_{k}=\frac{1}{2}\sum_{C(i)=k}\sum_{C(i')=k}d(x_{i},x_{i'})$$

where C(i) maps subject i to cluster k. The distance between two subjects i and i' is defined by

$$\text{Distance}=d(x_{i},x_{i'})=\sum_{j=1}^{p}{\left(x_{ij}-x_{i'j}\right)}^{2}$$

and x_i _denotes the vector of values of the first p principal components for subject i.

### Scoring Index (SI)

To identify the optimal number of clusters, we propose an algorithm and SI which evaluates the clustering accuracy, stability, and between cluster distance. The algorithm performs k-means clustering for each cluster size, k = 2, 3,...K, for M executions and computes the accuracy, stability and between cluster distance for each cluster size and execution. The rationale for incorporating these 3 measures into a scoring index is that the optimal cluster assignment should accurately allocate subjects to their respective cluster, should be stable from execution to execution of k-means and should maximize the distance between subjects allocated to different clusters. These three measures are computed in the observed data and are compared to those expected under random cluster allocation using permutation analysis. We summarize the gain in accuracy, stability and distance into a scoring index which is used to identify the optimal number of clusters. We discuss these steps in detail:

#### Cluster Accuracy

To measure the accuracy of each set of K clusters, we build a linear discriminant model using the cluster assignments from k-means, and then perform leave-one-out cross validation to estimate the accuracy to predict the cluster membership based on the linear discriminant model. If the clustering is accurate, we expect the linear discriminant model to accurately predict an individual's cluster membership. For each m, we compute the accuracy as the proportion of individuals assigned to the same cluster in cross validation as in k-means.

#### Cluster Stability

Since each execution of k-means clustering can produce different cluster assignments, we perform multiple executions (m = 1,..., M) of the clustering algorithm for each k, and measure the stability of the results. The stability will be worse for incorrect group sizes since k-means will be maximizing to a different local maximum each time, and thus low stability suggests that the number of clusters is not optimal. To measure the stability of the cluster assignments, we compute the Rand statistic [34] between the k-means cluster assignments for each number of clusters. The Rand statistic estimates the agreement between two sets of clusters by dividing the number of pairs of individuals in either the same cluster or in different clusters for both sets by the total number of pairs of individuals. Specifically, let *C*_1_, ..., *C*_*k *_and *X*_1_, ...., *X*_*k *_denote two sets of clusters generated in two executions of k-means for a fixed k. Let S be the number of pairs of subjects in the same cluster in both sets (for example subjects s and s' allocated both to *C*_*i *_and *X*_*j*_). Let D denote the number of pairs of subjects in different clusters for both sets (for example s in cluster *C*_*i *_and *X*_*i *_and s' in *C*_*j *_and *X*_*j*_), and T be the total number of pairs of subjects. Then the Rand statistic is defined as R = (S+D)/T. For each execution of k-means, we randomly choose 1 other execution of the same cluster size and compute the Rand statistic.

#### Distance: Between Cluster Scatter

At each cluster size k and execution m, the algorithm computes the normalized between-cluster distance, to monitor how distinct the clusters are from one another. This measure will always increase monotonically as the number of clusters increases. The between cluster distance provides information about the optimal number of clusters, however it does not necessarily provide a distinct number of clusters and does not measure the accuracy nor the stability of the cluster assignments.

#### Permutation

Accuracy, stability and between cluster distance are all dependent on the number of clusters and on the sample size in each cluster making it inappropriate to directly compare these measures across cluster sizes. For example, as the number of clusters increases, the accuracy decreases simply because it is more difficult to correctly predict an individual's cluster assignment with more available clusters (Figures 2, 4). Distance between clusters, on the other hand, will always increase as the number of clusters increases (Figures 2, 4). Therefore, we generate referent values for each k by randomly permuting the cluster labels generated in each execution of k-means and then compute the accuracy, stability and between cluster distance of the permuted cluster assignment. We then compute the relative gain in the observed accuracy, stability and between cluster distance to the permuted accuracy, stability and between cluster distance and combine the measures into a SI. Note that, since the permuted cluster assignments have the same number of clusters and subjects per cluster as the observed k-means cluster assignments, we are simulating these measures from the appropriate underlying distribution.

#### Scoring Index

To combine the measures of accuracy, stability and between cluster distance, the SI averages the relative gain in accuracy, relative gain in stability and relative gain in between cluster distance at each cluster size. Specifically, assume that for each execution m (m = 1,2,..., M) and cluster size k (k = 1,2,...,K) *A*_*O, m, k *_and *A*_*P, m, k *_are the observed and permuted accuracy, *B*_*O, m, k *_and *B*_*P, m, k *_are the observed and permuted between cluster distance and *S*_*O, m, k *_and *S*_*P, m, k *_are the observed and permuted stability. The SI is computed as:

$$SI_{k}=\frac{1}{M}\left[\sum_{m=1}^{M}\frac{\left(A_{O,m,k}-A_{P,m,k}\right)}{\left(1-A_{P,m,k}\right)}+\frac{\left(B_{O,m,k}-B_{P,m,k}\right)}{\left(1-B_{P,m,k}\right)}+\frac{\left(S_{O,m,k}-S_{P,m,k}\right)}{\left(1-S_{P,m,k}\right)}\right]$$

The SI ranges between 0 and 1. The optimal number of clusters is identified by the number of clusters that maximizes the SI. In some situations the differences between the SI can be relatively small for a range of cluster sizes. For the sake of parsimony, it is advisable to choose as the optimal solution the smallest number of clusters that has a mean within the 95% confidence interval of the maximum number of clusters. The confidence interval is computed in the following way. Since each score component falls between 0 and 1, a beta distribution is a reasonable distribution for each component. We used moment matching to estimate the parameters of the beta distribution and assume that the score components follow Beta distributions:

1. $\frac{\left(A_{O,m,k}-A_{P,m,k}\right)}{\left(1-A_{P,m,k}\right)}\sim beta\left(NA_{O,m,k}-NA_{P,m,k},N-NA_{O,m,k}\right)$, where N is the total sample size and *NA*_*O, m, k *_and *NA*_*P, m, k *_represent the number of subjects correctly assigned to their cluster for the observed and permuted assignments, respectively, for execution m and cluster size k.

2. $\frac{B_{O,m,k}-B_{P,m,k}}{1-B_{P,m,k}}\sim beta\left(SSB_{O,m,k}-SSB_{P,m,k},SST-SSB_{O,m,k}\right)$, where SST is the total distance between subjects measured by the informative PCs used for clustering and *SSB*_*O, m, k *_and *SSB*_*P, m, k *_are the sums of squares between clusters for the observed and permuted assignment, respectively, for execution m and cluster size k.

3. $\frac{\left(S_{O,m,k}-S_{P,m,k}\right)}{\left(1-S_{P,m,k}\right)}\sim beta\left(NS_{O,m,k}-NS_{P,m,k},NS-NS_{O,m,k}\right)$, where NS represents the total number of pairs of subjects and *NS*_*O, m, k *_and *NS*_*P, m, k *_are the number of concordant and discordant pairs for the observed and permuted cluster assignments, respectively, for execution m and cluster size k.

Then the variance of *SI*_*k *_is computed as

$$\begin{array}{l} Var(SI_{k})=\frac{1}{M^{2}}\frac{1}{9}[\sum_{m=1}^{M}[Var\left(\frac{A_{O,m,k}-A_{P,m,k}}{1-A_{P,m,k}}\right)\\ +Var\left(\frac{B_{O,m,k}-B_{P,m,k}}{1-B_{P,m,k}}\right)+Var\left(\frac{S_{O,m,k}-S_{P,m,k}}{1-S_{P,m,k}}\right)]]\\ =\frac{1}{M^{2}}\frac{1}{9}[\sum_{m=1}^{M}[\frac{\left(NA_{O,m,k}-NA_{P,m,k})(N-NA_{O,m,k}\right)}{{\left(N-NA_{P,m,k}\right)}^{2}(N-NA_{P,m,k}+1)}\\ +\frac{\left(SSB_{O,m,k}-SSB_{P,m,k})(SST-SSB_{O,m,k}\right)}{{\left(SST-SSB_{P,m,k}\right)}^{2}(SST-SSB_{P,m,k}+1)}\\ +\frac{\left(NS_{O,m,k}-NS_{P,m,k})(NS-NS_{O,m,k}\right)}{{\left(NS-NS_{P,m,k}\right)}^{2}(NS-NS_{P,m,k}+1)}]] \end{array}$$

and the 95% confidence interval is computed as the 2.5^th ^and 97.5^th ^quantile of a beta(α,β) where $\alpha =\frac{(1-SI_{k})SI_{k}^{2}}{Var(SI_{k})}-SI_{k}$, $\beta =\frac{\alpha (1-SI_{k})}{SI_{k}}$

**Figure 11:**
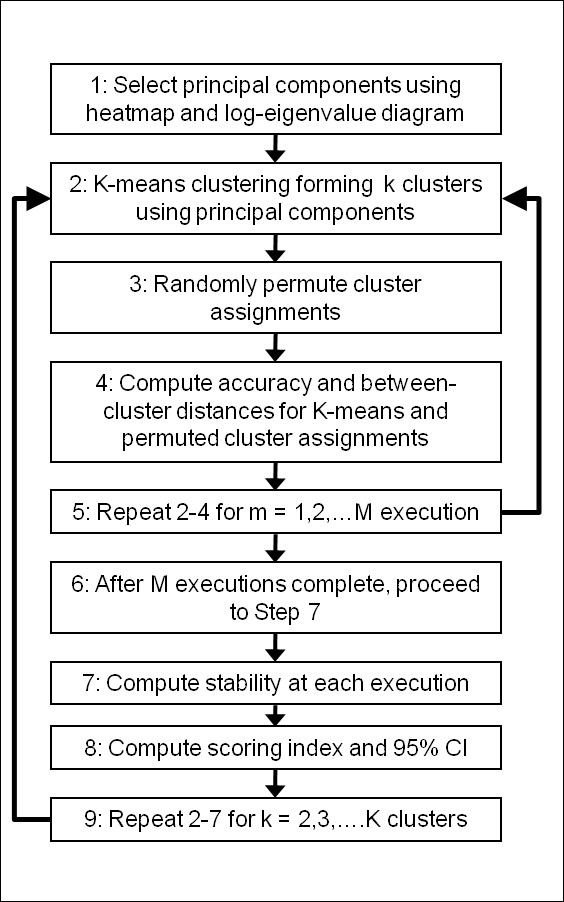
**Computation of Clustering Algorithm**. The flow chart outlines the steps of the algorithm. The optimal number of clusters is identified by the number of clusters that maximizes the scoring index. In some situations the scoring index can be relatively equal for a range of cluster sizes in which case it is advisable to choose the smallest number of clusters that has a SI within the 95% confidence interval of the maximum SI. We implement this heuristic in all examples.

The computation of the algorithm is summarized in Figure 11. In all examples, we used M = 100 and K = 30. A function for the clustering algorithm written in R and an example is provided as additional file 1. The run time for one thousand, 2 thousand and 3 thousand individuals with M = 100 and K = 30 is 13 minutes, 34 minutes and 69 minutes, respectively.

### Gap Statistic

The gap statistic [26] was computed for the HGDP Africans and the NECS using the gap() function of the SAGx package [35] in R [36]. The function is extremely computer intensive and thus we computed the gap statistic on 5 random cluster assignments, as computed by k-means, out of the total 100 executions for each cluster size. The statistic could not be computed on the HGDP African dataset for cluster sizes larger than 22 because at least 1 cluster contained only 1 subject.

### GWAS Analysis

All GWAS used a logistic regression model with an additive model for the SNP genotype and all SNPs had a call rate > 0.95 and MAF > 0.01. Analyses were performed using the software PLINK.

## Authors' contributions

NS developed and implemented the algorithm, conducted all the analysis and drafted the manuscript. SWH contributed to the implementation of the algorithm and the management of the data. CTB, TTP and MHS contributed data, helped with the evaluation of the analysis and the drafting of the manuscript. PS designed the study, helped to develop the algorithm and wrote the manuscript. All authors read and approved the final version of the manuscript.

## Supplementary Material

Additional file 1 **Implementation of clustering algorithm**. The zip file contains an R script with the implementation of the clustering algorithm, example files and help documentation. Click here for file

## References

[B1] Altshuler D Daly MJ Lander ES Genetic mapping in human disease Science 2008 322 5903 881 888 10.1126/science.1156409 18988837 PMC2694957

[B2] Marchini J Cardon LR Phillips MS Donnelly P The effects of human population structure on large genetic association studies Nat Genet 2004 36 5 512 517 10.1038/ng1337 15052271

[B3] Price AL Butler J Patterson N Capelli C Pascali VL Scarnicci F Ruiz-Linares A Groop L Saetta AA Korkolopoulou P Seligsohn U Waliszewska A Schirmer C Ardlie K Ramos A Nemesh J Arbeitman L Goldstein DB Reich D Hirschhorn JN Discerning the ancestry of European Americans in genetic association studies PLoS Genet 2008 4 1 e236 10.1371/journal.pgen.0030236 18208327 PMC2211542

[B4] Price AL Patterson NJ Plenge RM Weinblatt ME Shadick NA Reich D Principal components analysis corrects for stratification in genome-wide association studies Nat Genet 2006 38 8 904 909 10.1038/ng1847 16862161

[B5] Lee AB Luca D Klei L Devlin B Roeder K Discovering genetic ancestry using spectral graph theory Genet Epidemiol 2010 34 1 51 59 19455578 10.1002/gepi.20434 PMC4610359

[B6] Pritchard JK Stephens M Donnelly P Inference of population structure using multilocus genotype data Genetics 2000 155 2 945 959 10835412 10.1093/genetics/155.2.945 PMC1461096

[B7] Falush D Stephens M Pritchard JK Inference of population structure using multilocus genotype data: linked loci and correlated allele frequencies Genetics 2003 164 4 1567 1587 12930761 10.1093/genetics/164.4.1567 PMC1462648

[B8] Devlin B Roeder K Genomic control for association studies Biometrics 1999 55 4 997 1004 10.1111/j.0006-341X.1999.00997.x 11315092

[B9] Epstein MP Allen AS Satten GA A simple and improved correction for population stratification in case-control studies Am J Hum Genet 2007 80 5 921 930 10.1086/516842 17436246 PMC1852732

[B10] Kimmel G Jordan MI Halperin E Shamir R Karp RM A randomization test for controlling population stratification in whole-genome association studies Am J Hum Genet 2007 81 5 895 905 10.1086/521372 17924333 PMC2265648

[B11] Wang K Testing for genetic association in the presence of population stratification in genome-wide association studies Genet Epidemiol 2009 33 7 637 645 10.1002/gepi.20415 19235185

[B12] Hinds DA Stokowski RP Patil N Konvicka K Kershenobich D Cox DR Ballinger DG Matching strategies for genetic association studies in structured populations Am J Hum Genet 2004 74 2 317 325 10.1086/381716 14740319 PMC1181929

[B13] Guan W Liang L Boehnke M Abecasis GR Genotype-based matching to correct for population stratification in large-scale case-control genetic association studies Genet Epidemiol 2009 33 6 508 517 10.1002/gepi.20403 19170134 PMC2732762

[B14] Luca D Ringquist S Klei L Lee AB Gieger C Wichmann HE Schreiber S Krawczak M Lu Y Styche A Devlin B Roeder K Trucco M On the use of general control samples for genome-wide association studies: genetic matching highlights causal variants Am J Hum Genet 2008 82 2 453 463 10.1016/j.ajhg.2007.11.003 18252225 PMC2427172

[B15] Li JZ Absher DM Tang H Southwick AM Casto AM Ramachandran S Cann HM Barsh GS Feldman M Cavalli-Sforza LL Myers RM Worldwide human relationships inferred from genome-wide patterns of variation Science 2008 319 5866 1100 1104 10.1126/science.1153717 18292342

[B16] Terry DF Sebastiani P Andersen SL Perls TT Disentangling the roles of disability and morbidity in survival to exceptional old age Arch Intern Med 2008 168 3 277 283 10.1001/archinternmed.2007.75 18268168 PMC2895331

[B17] Sebastiani P Solovieff N Puca A Hartley SW Melista E Andersen S Dworkis DA Wilk JB Myers RH Steinberg MH Montano M Baldwin CT Perls TT Genetic Signatures of Exceptional Longevity in Humans Science 2010 20595579 10.1126/science.1190532

[B18] Campbell MC Tishkoff SA African genetic diversity: implications for human demographic history, modern human origins, and complex disease mapping Annu Rev Genomics Hum Genet 2008 9 403 433 10.1146/annurev.genom.9.081307.164258 18593304 PMC2953791

[B19] Patterson N Price AL Reich D Population structure and eigenanalysis PLoS Genet 2006 2 12 e190 10.1371/journal.pgen.0020190 17194218 PMC1713260

[B20] Tian C Plenge RM Ransom M Lee A Villoslada P Selmi C Klareskog L Pulver AE Qi L Gregersen PK Seldin MF Analysis and application of European genetic substructure using 300 K SNP information PLoS Genet 2008 4 1 e4 10.1371/journal.pgen.0040004 18208329 PMC2211544

[B21] Christensen K Johnson TE Vaupel JW The quest for genetic determinants of human longevity: challenges and insights Nat Rev Genet 2006 7 6 436 448 10.1038/nrg1871 16708071 PMC2726954

[B22] Aulchenko YS Ripatti S Lindqvist I Boomsma D Heid IM Pramstaller PP Penninx BW Janssens AC Wilson JF Spector T Martin NG Pedersen NL Kyvik KO Kaprio J Hofman A Freimer NB Jarvelin MR Gyllensten U Campbell H Rudan I Johansson A Marroni F Hayward C Vitart V Jonasson I Pattaro C Wright A Hastie N Pichler I Hicks AA Falchi M Willemsen G Hottenga JJ de Geus EJ Montgomery GW Whitfield J Magnusson P Saharinen J Perola M Silander K Isaacs A Sijbrands EJ Uitterlinden AG Witteman JC Oostra BA Elliott P Ruokonen A Sabatti C Gieger C Meitinger T Kronenberg F Doring A Wichmann HE Smit JH McCarthy MI van Duijn CM Peltonen L ENGAGE Consortium Loci influencing lipid levels and coronary heart disease risk in 16 European population cohorts Nat Genet 2009 41 1 47 55 10.1038/ng.269 19060911 PMC2687074

[B23] Sabatti C Service SK Hartikainen AL Pouta A Ripatti S Brodsky J Jones CG Zaitlen NA Varilo T Kaakinen M Sovio U Ruokonen A Laitinen J Jakkula E Coin L Hoggart C Collins A Turunen H Gabriel S Elliot P McCarthy MI Daly MJ Jarvelin MR Freimer NB Peltonen L Genome-wide association analysis of metabolic traits in a birth cohort from a founder population Nat Genet 2009 41 1 35 46 10.1038/ng.271 19060910 PMC2687077

[B24] Greene CS Penrod NM Williams SM Moore JH Failure to replicate a genetic association may provide important clues about genetic architecture PLoS One 2009 4 6 e5639 10.1371/journal.pone.0005639 19503614 PMC2685469

[B25] Hastie T Tibshirani R Friedman JH The elements of statistical learning: data mining, inference, and prediction 2009 2 New York: Springer

[B26] Tibshirani R Walther G Hastie T Estimating the Number of Clusters in a Data Set via the Gap Statistic Journal of the Royal Statistical Society. Series B (Statistical Methodology) 2001 63 2 411 423 10.1111/1467-9868.00293

[B27] Zhuang JJ Zondervan K Nyberg F Harbron C Jawaid A Cardon LR Barratt BJ Morris AP Optimizing the power of genome-wide association studies by using publicly available reference samples to expand the control group Genet Epidemiol 2010 34 4 319 326 10.1002/gepi.20482 20088020 PMC2962805

[B28] Cavalli-Sforza LL Menozzi P Piazza A The history and geography of human genes 1994 Princeton, N.J.: Princeton University Press

[B29] Balding DJ Nichols RA A method for quantifying differentiation between populations at multi-allelic loci and its implications for investigating identity and paternity Genetica 1995 96 1-2 3 12 10.1007/BF01441146 7607457

[B30] Holsinger KE Weir BS Genetics in geographically structured populations: defining, estimating and interpreting F(ST) Nat Rev Genet 2009 10 9 639 650 10.1038/nrg2611 19687804 PMC4687486

[B31] Purcell S Neale B Todd-Brown K Thomas L Ferreira MA Bender D Maller J Sklar P de Bakker PI Daly MJ Sham PC PLINK: a tool set for whole-genome association and population-based linkage analyses Am J Hum Genet 2007 81 3 559 575 10.1086/519795 17701901 PMC1950838

[B32] Peloso GM Timofeev N Lunetta KL Principal-component-based population structure adjustment in the North American Rheumatoid Arthritis Consortium data: impact of single-nucleotide polymorphism set and analysis method BMC Proc 2009 3 Suppl 7 S108 10.1186/1753-6561-3-s7-s108 20017972 PMC2795879

[B33] Jolliffe IT Principal component analysis 2002 2 New York: Springer-Verlag

[B34] Rand WM Objective Criteria for the Evaluation of Clustering Methods Journal of the American Statistical Association 1971 66 336 846 850 10.2307/2284239

[B35] Broberg P SAGx: Statistical Analysis of the GeneChip 2008 R package version 1.14.020298300

[B36] R: A language and environment for statistical computing 2008 R Development Core Team 2.7.0

[B37] Predominant Ethnic Group by Region http://www.eupedia.com/europe/maps_of_europe.shtml#ethnicities

