# Supplementary material for: Clustering by genetic ancestry using genome-wide SNP data
Source: BMC Genet. 2010 Dec 9;11:108. doi: 10.1186/1471-2156-11-108 (PMC3018397; doi:10.1186/1471-2156-11-108)
Supplement: Additional file 1 — Implementation of clustering algorithm. The zip file contains an R script with the implementation of the clustering algorithm, example files and help documentation. [file 1471-2156-11-108-S1.ZIP › Help Documentation.pdf]

These scripts provide a function to compute a scoring index to choose the optimal number of clusters as described by Solovieff et al. "Clustering by Genetic Ancestry"
NOTE: Install "MASS" package prior to running compute.scoring.index()

SCRIPTS

ComputeScoringIndex.R:	Contains function to compute scoring index: compute.scoring.index(pcs.for.clustering, max.cluster.size, number.executions = 100, data , additional.stats = FALSE, nstart.kmeans = 10, kmeans.iter = 2000)

FUNCTION INPUT

pcs.for.clustering	Principal Components to use for clustering
max.cluster.size	Maximum number of clusters to test – must be greater than 2
number.executions	Number of times to perform k-means (executions) -- 100 by default – must be greater than 2
data	Principal component data -- data frame in which the first column contains the subject's ID, 2nd column contains principal component 1, 3rd column contains principal component 2 etc.
additional.stats	If TRUE, then function will output Accuracy, Rand statistics, Between cluster distance and Scoring Index for each cluster size and execution -- FALSE by default
nstart.kmeans	parameter for kmeans clustering: number of random starts for k-means (see nstart in kmeans documentation) -- 10 by default
kmeans.iter	parameter for kmeans clustering: maximum number of iterations allowed in kmeans (see iter.max in kmeans documentation) -- 2000 by default

OUTPUT

Output provides the scoring index with the 95% confidence interval for each cluster size.
We advise choosing the cluster size that has a scoring index falling within the confidence interval of the maximum scoring index

If additional.stats = T is specified then function will also provide the accuracy, Rand statistics, between cluster distances and scoring index at each cluster size and execution

EXAMPLE DATA
contains the top 20 principal components and eigenvalues for a simulated dataset for 400 subjects and 4 clusters

example_data_principal_components.csv:	top 20 principal components for each subject
example_data_eigenvalues.csv:	Eigenvalues
RunExample.R	R script to compute the scoring index on the example data
